# Biodiversity of *Streptococcus thermophilus* Phages in Global Dairy Fermentations

**DOI:** 10.3390/v10100577

**Published:** 2018-10-22

**Authors:** Katherine Lavelle, Ines Martinez, Horst Neve, Gabriele A. Lugli, Charles M. A. P. Franz, Marco Ventura, Fabio dal Bello, Douwe van Sinderen, Jennifer Mahony

**Affiliations:** 1 School of Microbiology & APC Microbiome Ireland, University College Cork, Western Road, T12 YT20 Cork, Ireland; 115221728@umail.ucc.ie; 2 Sacco Srl, 22071 Cadorago, Italy; i.martinez@saccosrl.it (I.M.); F.DalBello@saccosrl.it (F.d.B.); 3 Department of Microbiology and Biotechnology, Max Rubner-Institut, 24103 Kiel, Germany; horst.neve@mri.bund.de (H.N.); charles.franz@mri.bund.de (C.M.A.P.F.); 4 Laboratory of Probiogenomics, Department of Chemistry, Life Sciences and Environmental Sustainability, University of Parma, 43124 Parma, Italy; gabriele.lugli@genprobio.com (G.A.L.); marco.ventura@unipr.it (M.V.)

**Keywords:** ecology, dairy fermentation, phage typing, genomics

## Abstract

*Streptococcus thermophilus* strains are among the most widely employed starter cultures in dairy fermentations, second only to those of *Lactococcus lactis*. The extensive application of this species provides considerable opportunity for the proliferation of its infecting (bacterio)phages. Until recently, dairy streptococcal phages were classified into two groups (*cos* and *pac* groups), while more recently, two additional groups have been identified (5093 and 987 groups). This highlights the requirement for consistent monitoring of phage populations in the industry. Here, we report a survey of 35 samples of whey derived from 27 dairy fermentation facilities in ten countries against a panel of *S. thermophilus* strains. This culminated in the identification of 172 plaque isolates, which were characterized by multiplex PCR, restriction fragment length polymorphism analysis, and host range profiling. Based on this characterisation, 39 distinct isolates representing all four phage groups were selected for genome sequencing. Genetic diversity was observed among the *cos* isolates and correlations between receptor binding protein phylogeny and host range were also clear within this phage group. The 987 phages isolated within this study shared high levels of sequence similarity, yet displayed reduced levels of similarity to those identified in previous studies, indicating that they are subject to ongoing genetic diversification.

## 1. Introduction

*Streptococcus thermophilus* is believed to have emerged at the beginning of human dairy activity, around 7000 years ago [1,2]. This species has links to streptococcal commensal species of the oral cavity, particularly *Streptococcus salivarius*; however, phylogenetic analysis of concatenated sequences of four genetic loci of *S. salivarius*, *Streptococcus vestibularis*, and *S. thermophilus* (component species of the salivarius group) demonstrated that *S. thermophilus* shared no common alleles with the two commensal species, defining it as a distinct species within the salivarius group [2]. Furthermore, multi-locus sequence typing of 27 *S. salivarius* and 27 *S. thermophilus* strains using identical primers identified 6% allelic variation among *S. salivarius* strains and typically <1% allelic variation among *S. thermophilus* strains. This low allelic variation among the *S. thermophilus* strains was somewhat surprising given the range of geographical isolation sources and the time span over which the strains had been isolated (over a period of 40 years) [2]. These results consolidate the suggestion that *S. thermophilus* populations emerged relatively recently and that limited diversity exists among this species. The limited genetic diversity of this species may also have implications for the diversity of phages that may infect its member strains.

For several decades, reports of phages infecting *S. thermophilus* were limited to two groups, termed the *cos* and *pac* groups. These were defined as distinct groups based on their modes of packaging (the use of cohesive ends or head-full packaging based systems) and the number of major structural proteins observed in SDS-PAGE profiles [3,4,5,6]. Members of the *cos* phage group are most frequently reported in studies of thermophilic dairy fermentations [7,8,9,10]. These are strictly virulent phages that possess isometric capsids and non-contractile tails of at least 200 nm [11]. In contrast, the *pac* phage group contains both virulent and temperate members, although the incidence of lysogeny in *S. thermophilus* is reported to be rather low at less than 10% [12,13]. Morphologically, the *pac* phages resemble *cos* phages with long non-contractile tails and isometric capsids. A comparative genome analysis of phages of these groups highlighted the genomic plasticity of dairy streptococcal phages, with modular genetic exchanges being a notable feature of the evolution of the genomes of these phages [6]. With the continuous isolation of only two (well characterized) groups of dairy streptococcal phages, there appeared to be a limited requirement for in-depth phage surveys of thermophilic dairy fermentations. However, two novel groups of *S. thermophilus* phages, that is, the 5093 and 987 groups, were identified in 2011 and 2016, respectively. Their genomes appear to have acquired genetic material from prophages of non-dairy streptococci in the case of the 5093 phages [14], and from the dairy starter bacterium *Lactococcus lactis* for the 987 phages [15]. The morphology of 5093 and 987 phages differs from those of *cos* and *pac* phages, as 5093 phages possess globular appendages attached to their tail tips, whereas the 987 phages possess considerably shorter tails than the other phage groups with a broad appendage at the tail tip that resembles the so-called “baseplate” of lactococcal P335 phages [6,9,14,15,16]. While genome sequencing revealed these two novel groups of dairy streptococcal phages, it is possible that they may have been in existence for some time. Indeed, electron micrographs of six phages of *S. thermophilus* isolated from Gruyère cheese in France in 1978 included images of a phage displaying the characteristics of 987 phages (short tails of 130 nm with broad baseplate appendages at the tail tip) [17]. Phages of these two groups have been subsequently isolated in another phage survey, highlighting an increasing prevalence of these phages [9,16]. This diversification of *S. thermophilus* phages highlights the need for continued efforts in the identification of phage populations in dairy processing facilities.

In the present study, 35 samples of whey derived from 27 thermophilic dairy fermentation facilities using distinct defined starter cultures were evaluated for the presence of phages against a panel of *S. thermophilus* strains. Members of all four *S. thermophilus* phage groups were identified and the genomes of representative isolates of each of the groups were sequenced in order to assess the extent of genetic diversity of phages in our collection.

## 2. Materials and Methods

### 2.1. Bacterial Strains

Fifty-two strains of *Streptococcus thermophilus* from the UCC culture collection were routinely grown at 42 °C from single colony or from bacterial stocks maintained at −20 °C (25% *w*/*v* glycerol) in M17 broth (Oxoid, UK) supplemented with 0.5% lactose (Sigma-Aldrich, St. Louis, MO, USA). Strains were characterised phenotypically as likely exopolysaccharide (EPS) producers in cases where “slimy” colony morphologies were observed on agar plates and/or where a ropy phenotype was observed after overnight incubation in broth. Secondly, the strains’ growth characteristics were also noted after overnight incubation as either sedimenting (S) or non-sedimenting (NS). The strains and their characteristics are listed in Table 1.

### 2.2. Bacteriophage Isolation from Whey and Enumeration

Thirty-five whey samples sourced from 27 distinct dairy fermentation facilities spanning ten countries on four continents were analysed for the presence of phages with lytic activity against 52 *S. thermophilus* strains. This was achieved using the standard double agar spot assay method [18], for which LM17 broth was supplemented with 0.25% glycine (Oxoid), 10 mM CaCl_2_, and 10 g/L (solid) or 4 g/L (semi-solid) agar-agar (Merck, Kenilworth, NJ, USA). Lytic phage activity was confirmed through the development of a clear zone of lysis within the bacterial lawn following overnight incubation at 42 °C. Phage positive samples were subsequently enumerated by plaque assays using the relevant whey–strain combination. Following overnight incubation, plaque morphology was assessed and isolates were deemed distinct based on size (small-medium-large) and/or halo formation. Individual plaque isolates were propagated on the relevant host strain in 10 mL LM17 broth supplemented with 10 mM CaCl_2_ at 42 °C, filtered through a 0.45 µm filter, and stored at 4 °C.

### 2.3. Characterisation of Bacteriophage Isolates

#### 2.3.1. Multiplex PCR typing

All bacteriophage isolates were subject to a multiplex PCR reaction to determine if they could be classified among the currently recognised groups of *S. thermophilus* phages, that is, *cos*, *pac*, 5093, and 987 groups. The primers used for the phage typing were based on those used in previous studies [9,10]. The primer sequences based on the *cos* phages were as follows: *cos*FOR (5′-ggttcacgtgtttatgaaaaatgg-3′) and *cos*REV (5′-agcagaatcagcaagcaagctgtt-3′) with an expected product size of 170 bp; those based on the *pac* phages were *pac*FOR (5′-gaagctatgcgtatgcaagt-3′) and *pac*REV (5′-ttagggataagagtcaagtg-3′) with an expected product size of 427 bp; those based on the 5093 phages were 5093FOR (5′-ctggctcttggtggtcttgc-3′) and 5093REV (5′-gcggcaaccatcttagaccag-3′) with an expected product size of 983 bp; and those based on the 987 phages were 987FOR (5′-ctaagcgtttgccactgtcag-3′) and 987REV (5′-gctgccgttgtttgaaaac-3′) with an expected product size of 707 bp. A PCR cycle of 95 °C for 10 min, followed by 30 cycles of (95 °C × 15 s, 55 °C × 30 s, 72 °C × 10 min), and a final single extension of 72 °C × 10 min was employed. The resulting amplicons were applied to a 1% agarose gel and visualized by UV transillumination.

#### 2.3.2. DNA Restriction Profiling

Restriction fragment length polymorphism profiling of select phage isolates was applied where distinction of phage isolates based on source or PCR typing could not be made. This was performed using *EcoRI* and *EcoRV* according to the manufacturer’s instructions (Thermo Fisher, Waltham, MA, USA). Then, 10 µL of restricted product was applied to a 1% agarose gel at 85 V for 45 min and visualized by UV transillumination.

#### 2.3.3. Host Range

Finally, to characterise the phages, a host range analysis of the individually propagated plaque isolates was performed. All bacteriophages were propagated to a titre of (at least) 10^7^ plaque forming units per ml (pfu/mL) and their host ranges were subsequently assessed on 51 additional strains from the collection listed in Table 1 first by spot assay, as described earlier. Presumptive positives were confirmed and enumerated by plaque assays and all assays were performed in triplicate.

### 2.4. Bacteriophage DNA Extraction

Bacteriophage DNA extraction was performed using methods adapted from the work of [19]. Phage DNA was extracted from 10 mL of fresh phage lysate (10^8^ pfu/mL), which was first treated with 1 μg/mL DNAse at 37 °C for 30 min, after which 50 µL of 0.5 M EDTA was added. After centrifugation at 13,200× *g* for 15 min, the lysate was transferred to a new tube, after which polyethylene glycol 8000 and sodium chloride were added to a final concentration of 10% and 0.5 M, respectively, and the resulting suspension was then incubated at 4 °C overnight. Subsequently, the suspension was centrifuged at 17,700× *g* for 15 min and the supernatant was removed. The PEG-precipitant was resuspended in 0.5 mL of TE buffer (pH 9.0) and treated with 40 μL of 20 mg/mL proteinase K for 20 min at 56 °C, and subsequently with 1.5% SDS for a further 10 min. Potassium acetate was added to a final concentration of 1 M, followed by incubation on ice for 20 min before centrifugation at 13,200× *g* for 10 min. The supernatant was then phenol/chloroform-extracted (25:24:1 phenol/chloroform/isoamyl alcohol, Sigma Aldrich) (at least) twice and the aqueous phase was precipitated with 2.5 volumes of ice-cold 96% ethanol and 0.1 volume of sodium acetate (pH 4.8). Subsequent to centrifugation, the pellet was washed in 70% ethanol and resuspended in 100 μL of TE buffer (pH 8.0). Where phage titre permitted (above 10^8^ pfu/mL), DNA extraction was performed using the Phage DNA Isolation Kit (Norgen Biotek Corp., Thorold, ON, Canada), according to the manufacturer’s instructions.

### 2.5. Genome Sequencing, Assembly, and Annotation

Thirty-nine isolates were selected for genome sequencing based on having unique/overlapping host ranges, restriction profiles, and geographical source of isolation. Genome sequencing of the selected phage isolates was performed using Illumina MiSeq sequencing technology (GenProbio, Parma, Italy). De novo sequence assemblies and automeated gene calling was performed using the MEGAnnotator pipeline [20] and assessed for predicted transfer RNA genes via tRNAscan-SE v1.2.1 [21]. Predicted open reading frames (ORFs) were determined via Prodigal v2.6 and Genemark.hmm [22]. A BLASTP [23] analysis was performed to assign functional annotations to the predicted ORFs (https://blast.ncbi.nlm.nih.gov/Blast.cgi). The proposed functional annotations were further confirmed by performing structural homology searches via HHpred [24] and querying the NCBI Conserved Domain Database (https://www.ncbi.nlm.nih.gov/Structure/cdd/wrpsb.cgi). The annotated genomes were inspected, edited, and finalised using the Artemis visualisation tool [25].

### 2.6. Genbank Accession Numbers

Genbank accession numbers for the sequenced phage genomes are presented in Table 2.

### 2.7. Comparative Genomic Analysis

Phage genomes were compared using nucleotide BLAST analysis of entire genomes, and comparison of the encoded individual proteins was performed by all-against-all, bi-directional BLAST alignment [26] with an alignment (or E-value) cut-off value of 0.0001 and greater than 50% identity across at least 50% of the amino acid sequence. Multiple alignment of nucleotide sequences of the phages isolated in this study, and of those of previously sequenced members of each of the four groups of *S. thermophilus* phages, was performed using ClustalW software. The alignment was employed to generate an unrooted phylogenetic tree using the “itol” software (http://itol.embl.de/), applying the neighbor-joining method.

### 2.8. Transmission Electron Microscopy

Phages SW11 (*cos*), SW13 (*pac*), SW16 (987), and SW27 (5093) were selected as representatives of the each of the four groups of *S. thermophilus* phages isolated in this study. Purification of the above-mentioned phages was achieved by double filtration of a high titre lysate (10^8–9^ pfu/mL) through a 0.45 µm filter. Adsorption of purified phages to freshly prepared carbon film floated from a freshly coated mica sheet and negative staining with 2% (*w*/*v*) uranyl acetate were performed as described previously [27]. The film was picked up with a 400-mesh copper grid (Agar Scientific, Essex, UK), and specimens were examined with a Tecnai 10 transmission electron microscope (FEI Thermo Fisher Scientific, Eindhoven, The Netherlands) operating at an acceleration voltage of 80 kV.

## 3. Results

### 3.1. Phage Screening of Whey Samples

Samples of whey from 27 factories in ten countries (Table 3) that were presumed to be phage-positive based on observations of slow acidification in the various cheese factories (and/or confirmations of phage contamination by the starter culture provider) were assessed for the presence of phages against a panel of 52 *S. thermophilus* strains. Of the 35 samples evaluated in this study, 25 were observed to cause lysis of at least one strain among the 52 tested strains in spot assays, while the starter culture provider identified 30 phage-positive samples against their strains (i.e., samples 1–5 and 34 were identified as phage-positive by the starter culture provider, while they were deemed phage-negative against our strain collection). This may indicate that the strains assessed in this study may not represent the full diversity required to capture the entire population of phages that may be present or that the numbers were too low at the time of processing. However, it is interesting that one sample (whey no. 17) was considered phage-negative by the starter culture provider, while it was found to contain phages against strains from our collection.

The presumptive phage-positive samples (25 samples) were confirmed by plaque assays and sample-specific titres on the identified host strains were observed between 10^2^ and 10^9^ pfu/mL (Table 3). This wide range of phage titres demonstrates the variation in phage load present in production samples derived from different factories and may be associated with (i) factory-specific practices such as hygiene practices including ineffective sanitization; (ii) starter-related issues including repeated application of preferred starter culture mixes; or (iii) the possibility that specific phages are more numerous than others within the same sample and against different strains.

Most of the phage-positive samples contained phages that could infect one to three strains (17 of 25 phage-positive samples, Table 3). However, approximately one-third of the phage-positive samples (8 of 25) contained phages that targeted four or more strains and, indeed, cases of phages targeting up to nine strains from a single sample were observed (Table 3). As the majority of *S. thermophilus* phages have reportedly narrow host ranges [9], it was inferred that the high number of strains infected by phages in these samples was because of the presence of multiple phages. This hypothesis was evaluated by propagating larger numbers of plaques from such samples than those where one to three samples displayed sensitivity (Table 3). Plaques were selected for propagation where observable differences in plaque morphology were observed or using different hosts for the phages from the same sample to increase the likelihood of capturing the diversity of phages that may be present in the samples. Using this approach, 172 phage isolates were propagated and characterized as will be described in more detail below.

### 3.2. Characterisation of Propagated Phage Isolates

The 172 phage isolates reported above were first characterized by multiplex PCR to establish the diversity of phage genotypes present in our collection. This PCR-based approach permitted the classification of the isolated phages as belonging to the *cos*, *pac*, 987, or 5093 groups of *S. thermophilus* phages [9,10]. Among the collection of 172 phages, 147 isolates were identified as *cos* phages, eleven as *pac* phages, nine as 987 phages, and five as 5093 phages. The distribution of these phage types among the tested samples is indicated in Table 3. In summary, 24 of the 25 phage-positive samples contained *cos*-type phages, seven contained *pac* phages, seven contained 987 phages, and four contained 5093 phages. While the majority of samples evaluated in this study (21 of 35) were sourced from Italian factories, *cos* phages were observed almost ubiquitously across all geographical locations, while the remaining phage types were shown to be associated with a confined selection of Italian, Turkish, and/or Argentine factories. Five samples (# 8, 9, 13, 14, and 21) harboured phages belonging to at least three of the four phage groups. Phages were not identified in the samples originating from Egypt and Congo, although just one sample was available from factories located in these countries.

Where (i) multiple phage isolates were propagated on a single host strain and/or (ii) where plaque morphologies of isolates propagated on the same host were highly similar, restriction fragment length polymorphism (RFLP) analysis was employed to identify unique isolates. In the vast majority of cases, the isolates from the same host and those exhibiting similar plaque morphology also displayed the same RFLP profile. To further characterize the phages within our collection, 100 isolates (composed of 79 *cos* phages, nine *pac* phages, eight 987 phages, and four 5093 phages) were selected for host range analysis based on having distinct primary host strains; geographical sources; plaque morphologies; phage typing reactions; and, where relevant, distinct RFLP profiles. Of the 52 strains tested, 23 were sensitive to infection by at least one of the 79 *cos* phage isolates; eight were sensitive to infection by at least one of the *pac* phages; and, interestingly, the 987 and 5093 phages infected only one strain each. Interestingly, at high titres, the 987 and 5093 phages could cross-infect the host strain of the opposite phage type (987 and 5093) at plaquing efficiencies of approximately 10^−4^ for the 987 phages and 10^−5^ for the 5093 phages. The highly confined host range of the 987 and 5093 phages among the collection of strains may underpin the relatively low frequency with which they are encountered in industry. While the *cos* and *pac* phages exhibit a typically narrow host range of one to three strains, a small number of isolates were observed to infect four or more strains (three *pac* isolates and one *cos* isolate). In order to define the variety of host-range interaction types present in the collection, the number of distinct host range profiles was assessed for the *cos* and *pac* isolates. For the *cos* phages, 39 distinct host range profiles were observed among the 79 isolates tested, while six distinct (although often overlapping) host ranges were observed for the eight *pac* phages.

During the characterization of our strain collection, the growth characteristics of each strain in broth were assessed. Subsequently, the growth type was defined as either “non-sedimenting” (NS) or “sedimenting” (S) depending on the appearance of the culture after overnight incubation. Host range analysis highlighted that while NS strains dominated our collection (42 of 52 strains), the primary selection of *pac* phages was more frequently observed on S strains, indicating that seven of the eight sequenced *pac* phages have a S type primary host. Furthermore, Table 1 highlights that all eight S strains were infected by at least one phage in this study, while approximately half of the NS strains (18 of 42) were sensitive to at least one phage in the collection. It was also noted that one of the three strains characterized as a possible EPS producer was sensitive to phage infection and thus it would appear that EPS production is not an impediment to infection among, at least some, *S. thermophilus* phages.

### 3.3. Phage Genome Sequencing

**Figure 1:**
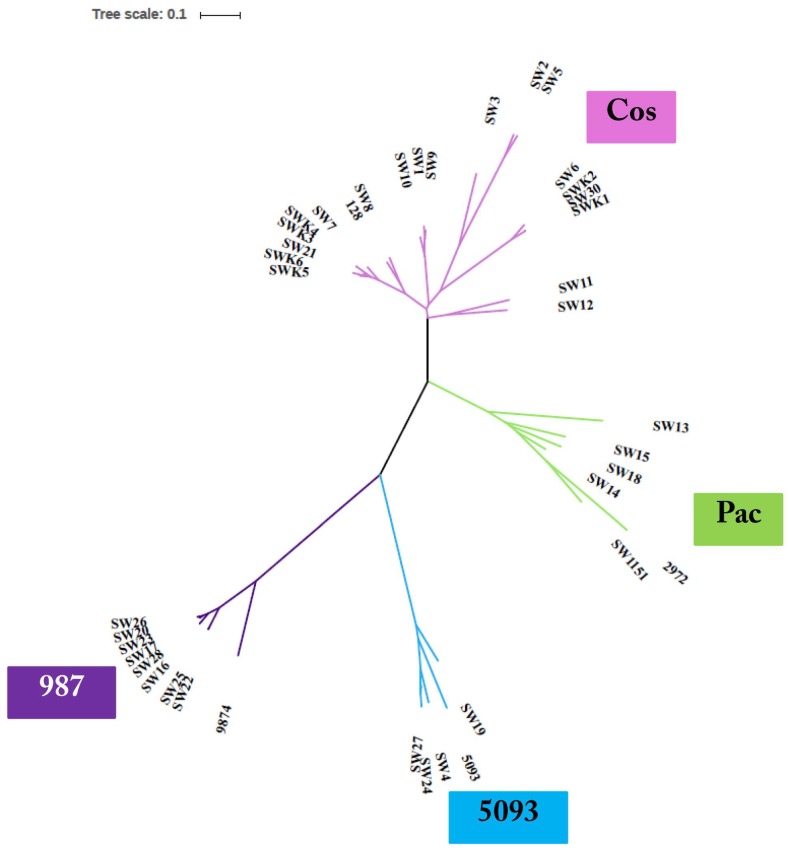
Unrooted phylogenetic tree of phage isolates and including representatives of each of the four described phage groups. The *cos* phages display most genetic diversity while the 987 and 5093 groups exhibit high genomic conservation irrespective of geographical background. Note: SW13, SW31, SW32, and SW33 are 100% identical. Therefore, this group of isolates is represented by SW13 on the phylogenetic tree for clarity.

Based on the outcome of the phage characterization assays detailed above, 39 representative phages (comprising nineteen *cos*, eight *pac*, four 5093, and eight 987 group phages) were selected for genome sequence analysis (Table 2). The genomes of the sequenced phages revealed genome lengths consistent with those sequenced in previous studies [7,8,9,16] with the 987 phages typically possessing the shortest genomes (31–32 kb) and those of the *cos* phages exhibiting the longest genomes (34–37 kb) (Table 2). Consistent with previous studies, limited genetic diversity was observed among the 5093 and 987 isolates sequenced in this study, while there is greater genomic diversity among the *cos* and *pac* isolates (Figure 1). More detailed information on each of the phage isolates is presented below.

#### 3.3.1. Genomic Diversity of the 987 Phages

**Figure 2:**
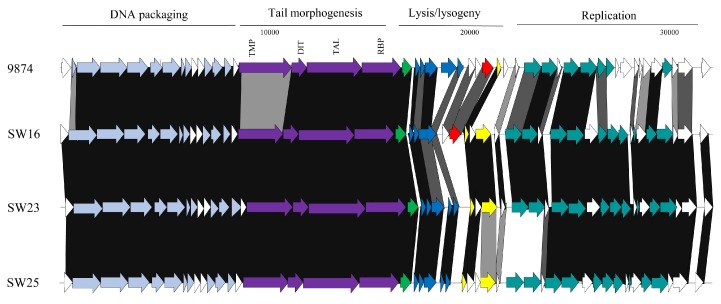
Genomic comparisons of representative 987 phages from this study and their most distant relative within the 987 group from previous studies, 9874. The functions of the gene modules are indicated above the diagram and correspond to the colour-coded arrows as follows: pale blue arrows represent genes encoding DNA packaging and capsid morphogenesis functions; purple arrows indicate tail adhesion/injection device components including the tape measure protein (TMP), Dit, Tal, and receptor binding proteins (RBPs); bright green arrow indicates a serine acetyltransferase-encoding gene; dark blue arrows indicate host lysis functions; red arrows indicate the presence of transposase-encoding genes; yellow arrows represent repressor/anti-repressor encoding genes; and teal green coloured arrows represent genes encoding predicted DNA replication functions. Where arrows are joined by a black shaded area, the represented gene products are between 90% and 100% similar at the aa level; dark grey indicates 70–89% similarity and light grey indicates 50–69% aa identity between the gene products. Unshaded regions represent those possessing less than 50% aa identity.

Phylogenetic analysis of the phage genomes highlighted the close relationship between the 987 group phages in particular (Figure 1), with 99% nucleotide identity over at least 90% of the genomes of these isolates based on BLASTn data. Comparing the genomes of the 987 group isolates in this study with those from previous studies [15,16], the query coverage drops to 70–85% of the genome. This reduction in sequence identity may be reflective of the fact that the phage isolates from the present study were all identified and propagated on a single host strain. Furthermore, the phages were all derived from Italian factories, albeit from six distinct factories using different (but possibly overlapping) starter cultures (Table 2). Therefore, while the diversity of 987 phages within a given study may be low, it may be speculated that there is greater diversity among the 987 group phages than previously considered. To provide genomic context to the variation of 987 phages isolated in different phage surveys, a comparative genomic analysis of representative isolates from the present study and those of the most distant member of the 987 group (phage 9874, based on BLASTn data) was carried out. This focused analysis demonstrated the high degree of synteny between the genomes of the 987 phages with no obvious genome shuffling. However, insertion/deletion events (indels) are observed among the studied 987 phage genomes and particularly so at the right end of the genome, which is purportedly involved in the genome replication functions. Indeed, at the left end of the genome, the only feature that displays reduced sequence similarity is the genomic region encoding the tail tape measure protein (TMP). Interestingly, the receptor binding proteins (RBPs) of these phages, which specify the host recognition functions of the phages, share greater than 95% aa identity, suggesting that host interactions among this group of phages are confined, which is consistent with the host range analysis (Figure 2).

#### 3.3.2. Genomic Diversity of the 5093 Phages

**Figure 3:**
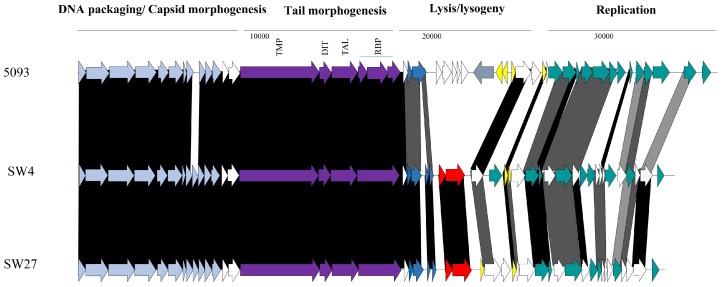
Genomic comparisons of representative 5093 phages from this study and the namesake of the group, 5093. The functions of the gene modules are indicated above the diagram and correspond to the colour-coded arrows as follows: pale blue arrows represent genes encoding DNA packaging and capsid morphogenesis functions; purple arrows indicate tail adhesion/injection device components including the TMP, Dit, Tal, and RBPs; dark blue arrows indicate host lysis functions; red arrows indicate the presence of transposase-encoding genes; yellow arrows represent repressor/anti-repressor-encoding genes; and teal green coloured arrows represent genes encoding predicted DNA replication functions. Where arrows are joined by a black shaded area, the represented gene products are between 90% and 100% similar at the aa level; dark grey indicates 70–89% similarity and light grey indicates 50–69% aa identity between the gene products. Unshaded regions represent those possessing less than 50% aa identity.

Similar to the 987 phages, limited genetic diversity was observed among the 5093 isolates (Figure 1), with 99% nucleotide identity across at least 69% of the phage genomes of isolates within the study and over 70% of the genome of the namesake of the phage group (5093) [14]. The majority of genetic variation is observed at the right end of the genomes (in the replication region) with modular acquisitions or smaller indels composed of single/small numbers of genes (Figure 3). As with the 987 group phages, the RBPs of these phages exhibit high sequence similarity, which, in this case, is also indicative of a narrow host range within this group of phages. Three of the four sequenced isolates originate from Italian factories, while one was isolated from an Argentinian whey sample. Therefore, location-specific genotypes or host range profiles cannot be assigned to these phages because all four phages infect the same host and their genomes share high levels of sequence similarity irrespective of geographical source. Despite the overall similarity, it is noteworthy that a certain level of genome plasticity is observed. This indicates that increased genetic diversity will be observed when additional isolates of this group will be sequenced in the future.

#### 3.3.3. Genomic Diversity of the *pac* Phages

**Figure 4:**
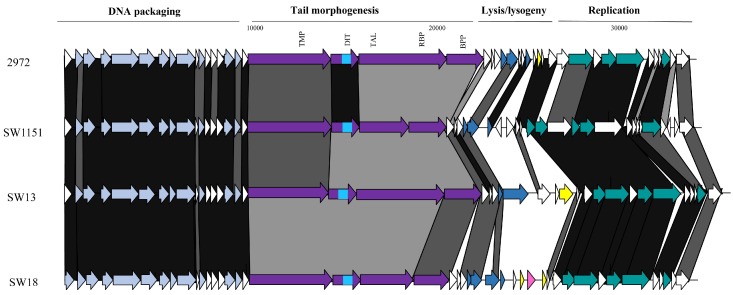
Genomic comparisons of representative *pac* phages from this study and the reference *pac* phage, 2972. The functions of the gene modules are indicated above the diagram and correspond to the colour-coded arrows as follows: pale blue arrows represent genes encoding DNA packaging and capsid morphogenesis functions; purple arrows indicate tail adhesion/injection device components including the TMP, Dit, Tal–RBP, and baseplate proteins (BPP); pink arrows indicate the presence of lipoprotein-encoding genes; yellow arrows represent repressor/anti-repressor encoding genes; and teal green coloured arrows represent genes encoding predicted DNA replication functions. The bright blue area within the arrow representing the Dit-encoding genes represents the region that encodes a carbohydrate binding domain. Where arrows are joined by a black shaded area, the represented gene products are between 90% and 100% similar at the aa level; dark grey indicates 70–89% similarity and light grey indicates 50–69% aa identity between the gene products. Unshaded regions represent those possessing less than 50% aa identity.

Despite the limited number of *pac* phages isolated and sequenced in this study, there appears to be a significant degree of genetic diversity among these isolates. The phages were isolated from five distinct whey samples from Italy (four) and Turkey (one). Given that the eight sequenced isolates represent four host range profile groups, it is perhaps unsurprising that the region encoding the host adhesion device (incorporating TMP, Dit, and the fused Tal–RBP proteins) is among the most significant areas of sequence dissimilarity, both within the isolates from this study as well as in comparison with the well-studied and related *pac* phages (including phage 2972 used as a comparator in this study; Figure 4). The genomic regions specifying the (remnants of the) lysogeny module and the replication functions represent the other dominant regions of divergence (Figure 4). In these regions, a number of indels and individual gene products displaying sequence divergence can be identified, highlighting the plasticity of their genomes and the phage-specific nature of these modules. One of the most striking findings from the analysis of the *pac* phage genomes was the isolation of identical phages (SW13, SW31, SW32, and SW33) in four distinct whey samples in three geographically disparate locations (Turkey, Italy, and Argentina). However, these isolates all exhibit a broad host range infecting five strains, which may underpin the success of this phage in various fermentations.

#### 3.3.4. Genomic Diversity of the *cos* Phages

**Figure 5:**
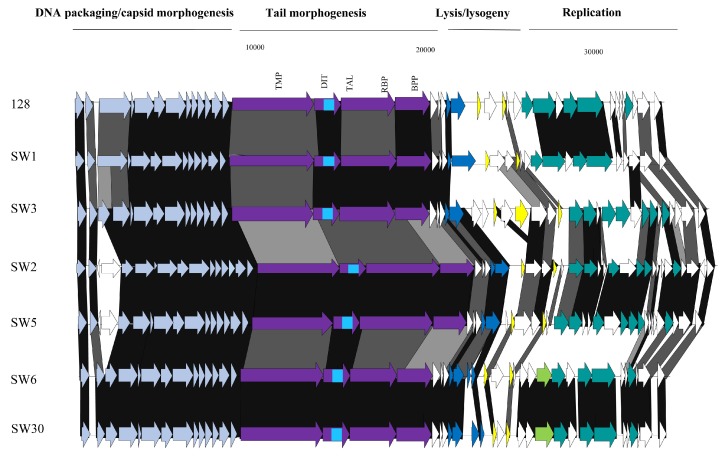
Genome comparisons of representative *cos* phages from this study and in comparison with phage 128 from a previous study [7]. The functions of the gene modules are indicated above the diagram and correspond to the colour-coded arrows as follows: pale blue arrows represent genes encoding DNA packaging and capsid morphogenesis functions; purple arrows indicate tail adhesion/injection device components including the TMP, Dit, Tal–RBP, and baseplate proteins (BPP); yellow arrows represent repressor/anti-repressor encoding genes; lime green arrows represent type III restriction endonuclease R subunit-encoding genes; and teal green coloured arrows represent genes encoding predicted DNA replication functions. The bright blue area within the arrow representing the Dit-encoding genes represents the region that encodes a carbohydrate binding domain. Where arrows are joined by a black shaded area, the represented gene products are between 90% and 100% similar at the aa level; dark grey indicates 70–89% similarity and light grey indicates 50–69% aa identity between the gene products. Unshaded regions represent those possessing less than 50% aa identity.

Among *S. thermophilus* phages, the *cos* group is most frequently encountered in the industry and is the most extensively studied. The current study proved no exception in that 147 of 172 isolates were shown to belong to the *cos* group (of which 19 were sequenced) (Table 2). The genomes of the *cos* phages described in this study display the greatest genetic diversity of all four *S. thermophilus* phage groups. The 19 sequenced isolates each represent a distinct (and in some cases overlapping) host range profile and the genetic diversity observed in the phylogenetic tree (Figure 1) and the comparative genomic analysis (Figure 5) further corroborates the diversity of geographical sources (nine Italian factories; two Turkish factories; and one factory in Mexico, France and Austria) and host interactions of these phages. The right end of the genomes encoding the replication functions display significant diversity (Figure 5) with an array of modular acquisitions and variations observed. The region specifying the adhesion device displays significant sequence variation, although the gene order is perfectly conserved among all sequenced members of the group. The adhesion device of the *cos* type phages incorporates (parts of) the TMP, Dit, Tal–RBP and additional accessory proteins. The TMP is responsible for phage length determination of phages [28], while roles in genome injection [29] and cell wall degradation to facilitate efficient infection [30,31] have also been identified in phages infecting a range of hosts. The well-studied *cos* phage DT1 was previously observed to harbor a CHAP (cysteine histidine-dependent aminohydrolase/peptidase) domain and an SLT (soluble lytic transglycolase) domain at the carboxy terminus of its encoded TMP. All sequenced members of the *cos* group in this study also harbor these domains at the C-terminus of their respective TMPs. The presence of these lytic domains within the TMP appears to be unique to the *cos* phages as this was not observed among the TMPs of any of the other phage groups. The Dit proteins of the *cos* and *pac* phages contain additional domains relative to those of other phage groups, which, in *Lactobacillus* and lactococcal phages, constitutes an “evolved” Dit [30,32]. These additional domains are comprised of carbohydrate binding domains and, given that several adhesion device proteins were previously proposed to be involved in host binding and infection, it is plausible that such domains may improve the adsorption capabilities of phages [31]. The Tal–RBPs of these phages are genetically diverse, as might be expected if this component is indeed the primary determinant of host specificity. The RBPs of these phages will be discussed in more detail below. Given the intricate and diverse range of phage–host interactions within the *cos* isolates, it would be expected that the components of the adhesion device exhibit sequence divergence.

#### 3.3.5. Phage-Encoded RBPs and Host Range

**Figure 6:**
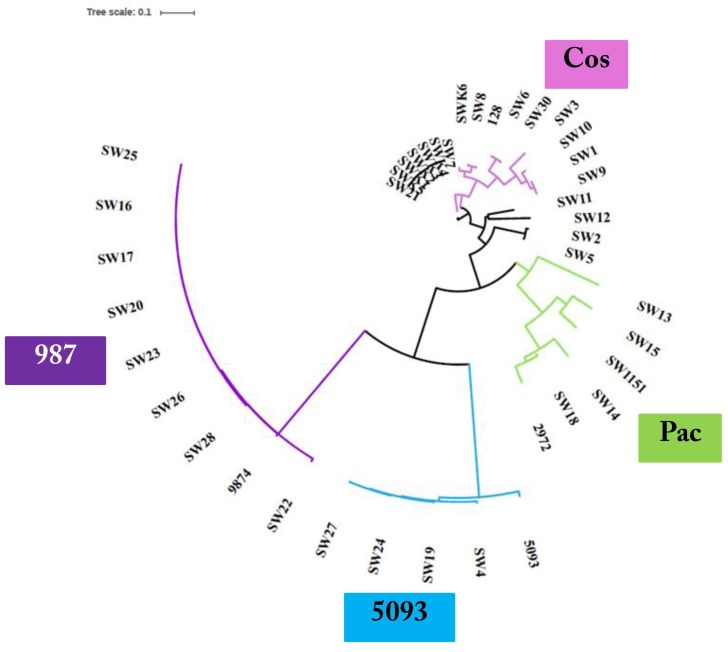
Phylogenetic analysis of the amino acid sequences of the encoded RBPs of the phages sequenced as part of this study. The Tal–RBP of SW13, SW31, SW32, and SW33 are 100% identical. Therefore, SW13 is used as a representative of these four phage-encoded Tal–RBPs for clarity.

The sequences of the Tal–RBPs of all phages sequenced as part of this study were compared and reference sequences for each of the four groups were included (as per the overall comparative genomic analysis detailed above). The RBPs of the 987 and 5093 groups were almost identical within their respective groups (Figure 6). This is consistent with the finding that all 5093 isolates infected only UCCSt89, while all of our 987 isolates exclusively infected UCCSt97. The Tal–RBP sequences of the *pac* phages isolated from the Italian samples (four phages) group quite closely and infect three distinct strains, while the isolate from the Turkish sample (SW13) appeared to be distinct (and represent those of the identical isolates SW31–SW33). The latter phage infects five *S. thermophilus* strains from our collection, which is a relatively broad host range by comparison with the majority of dairy streptococcal phages. Phages SW31, SW32, and SW33 are identical to SW13, and thus also exhibit the same host range. Therefore, there are five genetic lineages among the *pac* isolates and, while the primary/propagating hosts of four of these five distinct *pac* phages are unique, their host range profiles contain several incidences of one or more overlapping strains preventing a clear link between the RBP phylogeny and host specificity from being derived. Interestingly, the Tal–RBPs of the *cos* phage isolates display a very clear correlation between Tal–RBP phylogeny and primary host and/or host range. For example, SW2 and SW5 share an identical primary host strain (UCCSt86); SW1, SW9, and SW10 share UCCSt23 as their primary host; and SW6 and SW30 both infect strain UCCSt95, among other similarly related groups. This corroborates the primary role of the Tal–RBP of *cos* phages in host recognition and binding.

### 3.4. Morphological Analysis of Phage Isolates

**Figure 7:**
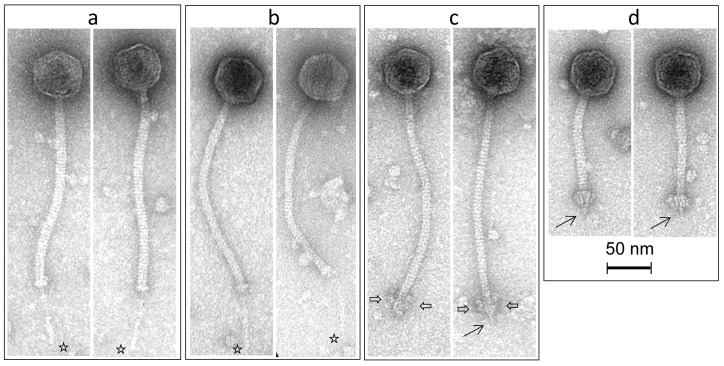
Transmission electron micrographs of representative phages belonging to the *cos* (SW11—panel A); *pac* (SW13—panel B); 5093 (SW27—panel C); and 987 groups (SW16—panel D). The presence of long tail fibers is indicated by a star (panels (**a**,**b**)); globular appendages at the distal end of the 5093 phage are indicated by a double-sided arrow (panel (**c**)); and short tail fibers are indicated by a single-lined arrow (panels (**c**,**d**)).

A representative of each of the four groups of phages (*cos*, *pac*, 987, and 5093) was selected for purification and subsequent morphological analysis by transmission electron microscopy. All four phages were observed to exhibit the typical characteristics of their groups. The diameter of the capsids of the phages ranges between 55 and 59 nm. SW11 (*cos* phage) exhibits a tail of 228.1 ± 9.1 nm (*n* = 17) with a feather-like tail fibre appendage of 72.2 ± 5.2 nm (*n* = 11) protruding through the tip. This remarkably delicate structure has been observed in previous members of this group. SW13 (*pac* phage) possesses a long non-contractile tail of 231.9 ± 5.5 nm (*n* = 15) and also appears to possess a short tail fibre of 79.5 ± 10.1 nm (*n* = 8) at the distal end of the tail. SW27 (5093 phage) exhibits the typical globular appendages attached to the distal end of the tail—a feature that appears to be typical of the 5093 phages. The tail of this phage is 242.1 ± 6.9 nm (*n* = 23) with a tail tip appendage width of 27.4 ± 2.6 nm (*n* = 23) and length of 19.5 ± 2.7 nm (*n* = 23). SW16 (987 phage) possesses a relatively short tail of 124.9 ± 3.2 nm (*n* = 18) and a broad adhesion device at the tail tip (22.1 ± 2.7 nm; *n* = 18) that is more akin to the lactococcal P335 phages than to those of other *S. thermophilus* phages and that is typical of members of the 987 phage group, owing to the apparent ancestral acquisition of lactococcal genetic elements (Figure 7). SW16 was also observed to possess a discrete tail fibre of 14.2 ± 1.8 nm (*n* = 6).

## 4. Discussion

In this study, 172 phages were isolated from 25 phage-positive samples among the 35 whey samples tested. Microbiological and molecular characterization of these isolates facilitated the identification of 39 distinct isolates that were selected for genome sequence analysis. The samples were derived from 27 factories in 10 countries using distinct defined cultures incorporating different, but potentially overlapping, strains of *S. thermophilus* and adjunct cultures in some cases sourced from the same starter culture provider. Therefore, perhaps it is possible to derive that the diversity of phages observed in this study is a reflection of the genetic and phenotypic diversity of starter cultures applied in the various factories as supplied by the starter culture provider.

Thermophilic dairy fermentations are reliant on the application of *S. thermophilus* strains, albeit often in partnership with adjunct cultures such as *L. helveticus* or *L. delbrueckii* ssp. *bulgaricus*, among others. Until recently, only two groups of dairy streptococcal phages were known to be problematic in industrial fermentations. However, since the first reports of the novel 5093 and 987 phage groups in 2011 and 2016, respectively, it has become clear that there may be a greater diversity of *S. thermophilus* than previously considered. Furthermore, given the relative homogeneity of dairy streptococci, it seemed unlikely that significant diversity exists beyond the well-studied *cos* and *pac* groups. It seems that valuable lessons may be learned from the archives, as morphological analysis of phages isolated from Gruyère cheese in 1979 demonstrated the presence of a phage with short tail and broad baseplate, seemingly atypical features for dairy streptococcal phages [17]. It was not until almost 40 years later that these phages were once again isolated and classified as the 987 phages [15]. In the present study, we report the isolation and characterization of members of each of the four groups of *S. thermophilus* phages. It is clear from host range profiling that the 5093 and 987 phages each primarily infect a single strain and that at high titres, they cross-infect the primary host strain of the other group. The remarkable specificity of these phages is likely the reason underpinning their infrequent isolation and suggests that they are associated with the application of very specific strains in dairy fermentations without which they cannot proliferate. The cross-infection of hosts between these “rare” phage groups suggests that the host strains of these two phage groups may often be partnered or rotated in certain production regimes, allowing them to adapt to infect the host of the opposite group or that similar receptor motifs are present in both strains. It may also be indicative of the phages’ ability to overcome phage-resistance mechanisms that may be present on the secondary host strain, including restriction/modification and CRISPR-Cas systems. While the CRISPR spacer arrays of these strains were not examined in the context of the present study, the CRISPR1 and CRISPR3 systems of dairy streptococci are known to be highly active in acquiring spacers upon exposure to phages [7].

While limited genetic diversity is currently observed among the 987 and 5093 groups, it is clear that isolates from different studies exhibit reduced sequence similarity; therefore, it is likely that these phages will adapt and continue to evolve. The relatively low numbers of sequenced members of these two groups in comparison with the *cos* and *pac* groups prevent meaningful conclusions as to the extent of diversity of these phages from being drawn. However, given the recent increase in frequency of isolation of these phages, it is very likely that this number will increase considerably over the coming years, permitting meaningful evaluations of these phage groups to be performed. Furthermore, the recent development of PCR-based classification tools for the 987 and 5093 phages will enhance the efficiency of identification of new members of these groups in future studies [9].

As consumer demands and market needs change continuously, producers implement specific strains/starter culture blends that impart the desired organoleptic characteristics. The (modest) increase in the frequency of isolation of 987 and 5093 phages in recent thermophilic phage surveys is an interesting observation. It is tempting to speculate that the (currently) preferred organoleptic properties of dairy products are influencing starter/strain selections, which, in turn, may selectively enhance the proliferation of certain phages such as the 987 and 5093 groups.

The specificity of phage–host interactions is primarily dictated by phage-encoded Tal–RBPs [33], although additional phage-encoded factors may be involved [31,34]. In the present study, a clear correlation was observed between host range and Tal–RBP phylogeny of the *cos* phages in a manner reminiscent of the lactococcal 936 group phages [33,35]. This correlation supports previous studies that report that the Tal–RBP is the primary determinant of host recognition and binding in *cos* phages [8,36]. The RBPs of the 987 and 5093 phages (these phage groups do not have fused Tal–RBP functions) have recently also been experimentally confirmed; however, as all isolates of these groups in this study infected the same strains, it was not possible to define if other factors contribute to host recognition beyond the RBP [8,9,15]. It was previously remarked that *cos* and *pac* phages typically exhibit limited overlap in the host range [37], a finding mirrored in the present study. A Tal–RBP–host range relationship could not be clearly discerned for the *pac* isolates in this study. This may be because of the requirement of additional components of the adhesion device for effective recognition and binding. An interesting observation is the identification of so-called “evolved” Dits encoded by the *cos* and *pac* phages. Such evolved Dit proteins have been identified in phages of *Lactobacillus* and *Lactococcus*, and in the case of *Lactobacillus casei* phages, these components were observed to harbor carbohydrate domains that were capable of replacing RBP functions. The diversification of the Dit proteins of dairy streptococcal *cos* and *pac* phages exemplifies the continuous evolution of the adhesion devices of phages infecting lactic acid bacteria in order to adapt to new hosts or to provide selective advantages in increasing the binding frequency/efficiency in order to ensure the continued success and proliferation of a given phage. From an industrial perspective, this diversification represents a constant threat to production practices. While plant design, personnel training, and sanitisation regimes may be improved and controlled, it is nearly impossible to remove phages from a plant environment. Therefore, regular phage monitoring within factories or by starter culture providers and wide-ranging phage surveys, such as the one reported here, are vital to ensure that we continue to understand the phage landscape in the dairy processing environment. This will support the production of consistent dairy fermented products with the desired organoleptic properties.

The first reported members of the 987 group were identified because the isolates could not be typed using the multiplex PCR designed to classify *cos* and *pac* phages [15]. Subsequently, primers were incorporated into the multiplex PCR system to include those that would detect 5093 and 987 phages [9]. In future screens for potentially novel *S. thermophilus* phages, those that cannot be typed by the updated PCR set-up will be readily identified and selected for further analysis, thus refining and expediting diversity analyses. Such tools are essential to rapidly detect and classify of problematic phages in a given factory. In our study, the multiplex PCR successfully typed each of the 172 isolates, indicating that the primers are effective in classifying recent global isolates, thus validating their usefulness in practice.

*S. thermophilus* strains are renowned for their lack of genetic diversity; however, phenotypically, some diversity is observed with some strains being ropy and slimy, characteristics generally associated with EPS production. In contrast, other strains display a sedimenting phenotype, while others grow homogeneously throughout the growth medium. These growth characteristics are indicative of varying surface properties and structures among *S. thermophilus* strains. Such phenotypic characteristics are often used as selectable traits in the dairy industry, for example, EPS production and higher viscosity would be desirable in strains applied to yogurt production. The majority of strains (42 of 52) in our collection exhibit an NS phenotype and 18 of these strains were susceptible to infection by at least one phage isolate. Eight strains displayed an S phenotype, with a further two exhibiting features of both sedimentation and growth in the medium. All ten of these strains were infected by phages within the isolated bank. It is interesting to note that all S strains were shown to be sensitive to phage infection, although it is impossible to conclude if S strains may typically be more phage sensitive than NS strains; however, it may be an interesting feature that may readily be assessed in an industrial context where much larger strain collections are available.

The isolation of members of all four *S. thermophilus* phage groups in this study is a reminder of the ongoing requirement for phage monitoring programs to understand the risk factors that may be present in any given factory and against any panel of starter cultures. Through such studies, tools to rapidly detect these industrial parasites may be developed, providing the opportunity to limit the proliferation of phages and to implement suitable strategies to ensure the consistent production of end-products with the desired characteristics and quality. This is imperative to the continuing success of the dairy fermentation industry.

## Figures and Tables

**Table 1:** Bacterial strains used in this study and their phenotypic characteristics.

S. thermophilus Strain	Strain Characteristics *	Phage Sensitive
STP1	NS	+
UCCSt57	NS	+
UCCSt13	NS	+
UCCSt82	NS	+
UCCSt63	NS	+
UCCSt23	NS	+
UCCSt83	NS	+
UCCSt84	NS	+
UCCSt85	NS	+
UCCSt86	NS	+
UCCSt87	NS	+
UCCSt88	NS	+
UCCSt89	NS	+
UCCSt90	NS	+
UCCSt91	NS	+
UCCSt9	NS	+
UCCSt97	NS	+
UCCSt101	NS	+
UCCSt53	NS	+
STP2	NS	-
UCCSt14	NS	-
UCCSt61	NS	-
UCCSt62	NS	-
UCCSt65	NS	-
UCCSt98	NS	-
UCCSt67	NS	-
UCCSt68	NS	-
UCCSt55	NS	-
UCCSt57	NS	-
UCCSt28	NS	-
UCCSt29	NS	-
UCCSt64	NS	-
UCCSt66	NS	-
UCCSt73	EPS+	-
UCCSt69	EPS+	+
UCCSt54	EPS+	-
UCCSt99	NS	-
UCCSt8	NS	-
UCCSt21	NS	-
UCCSt33	NS	-
UCCSt22	NS	-
UCCSt10	NS	-
UCCSt92	S	+
UCCSt51	S	+
UCCSt93	S	+
UCCSt12	S	+
UCCSt94	S	+
UCCSt50	S	+
UCCSt95	S	+
UCCSt96	S	+
UCCSt31	S/NS	+
UCCSt1	S/NS	+

* S indicates a strain that displays a sedimenting phenotype after overnight growth in broth; NS indicates a non-sedimenting phenotype after overnight growth in broth; S/NS indicates those strains for which large pellets and homogeneous growth were observed in parallel; and exopolysaccharide (EPS)+ indicates those strains exhibiting a slimy colony morphology typical of EPS-producers.

**Table 2:** Summary of the genome characteristics of the sequenced *S. thermophilus* phage genomes. ORF—open reading frame.

Phage	Whey Sample	Country of Origin	Host Strain	# Strains Infected ^†^	Type	Genome Size (bp)	*#* ORFs	G&C%	Genbank Accession No.
SW1	16	Italy	UCCSt23	1	*cos*	34,821	38	38.85	MH892352
SW2	13	Italy	UCCSt86	1	*cos*	37,222	47	38.81	MH892353
SW3	6	Italy	UCCSt83	1	*cos*	37,181	45	38.64	MH892354
SW5	14	Italy	UCCSt86	1	*cos*	36,836	47	38.78	MH973661
SW6	8	Italy	UCCSt95	1	*cos*	34,276	42	38.64	MH892351
SW7	12	Italy	UCCSt84	2	*cos*	35,917	42	39.11	MH973662
SW8	31	Mexico	UCCSt88	1	*cos*	33,930	41	39.11	MH892357
SW9	17	Italy	UCCSt23	2	*cos*	33,999	38	38.71	MH892358
SW10	32	France	UCCSt23	1	*cos*	34,336	39	38.76	MH892359
SW21	11	Italy	UCCSt82	3	*cos*	35,351	39	38.78	MH892356
SW30	35	Austria	UCCSt95	1	*cos*	34,158	41	38.46	MH892375
SW12	29	Turkey	UCCSt96	1	*cos*	35,382	43	38.75	MH892361
SW11	29	Turkey	UCCSt92	1	*cos*	36,250	42	38.23	MH892360
SWK2	9	Italy	UCCSt63	3	*cos*	36,594	42	39.30	MH892378
SWK6	10	Italy	UCCSt63	3	*cos*	34,999	38	39.01	MH892382
SWK1	9	Italy	UCCSt63	3	*cos*	36,089	43	39.17	MH892377
SWK3	31	Mexico	UCCSt63	3	*cos*	35,443	41	38.72	MH892379
SWK5	21	Italy	UCCSt63	2	*cos*	35,849	39	38.90	MH892381
SWK4	28	Turkey	UCCSt63	3	*cos*	35,341	41	38.69	MH892380
SW31 *	12	Italy	UCCSt50	5	*pac*	36,587	41	39.24	MH892383
SW32 *	14	Italy	UCCSt50	5	*pac*	36,587	41	39.24	MH892384
SW33 *	27	Argentina	UCCSt50	5	*pac*	36,587	41	39.24	MH892385
SW13 *	29	Turkey	UCCSt50	5	*pac*	36,587	41	39.24	MH892362
SW14	9	Italy	UCCSt96	2	*pac*	35,913	46	39.87	MH892363
SW15	12	Italy	UCCSt96	1	*pac*	34,289	44	39.39	MH892364
SW18	8	Italy	UCCSt93	2	*pac*	34,825	44	39.43	MH892366
SW1151	14	Italy	UCCSt10	1	*pac*	35,056	45	39.60	MH892376
SW16	7	Italy	UCCSt97	1	987	32,106	45	36.95	MH892350
SW17	8	Italy	UCCSt97	1	987	32,005	46	36.84	MH892365
SW20	9	Italy	UCCSt97	1	987	32,567	46	36.81	MH892368
SW22	13	Italy	UCCSt97	1	987	31,738	45	37.35	MH892369
SW23	13	Italy	UCCSt97	1	987	31,422	45	37.03	MH892370
SW25	14	Italy	UCCSt97	1	987	32,013	46	37.20	MH892371
SW26	15	Italy	UCCSt97	1	987	31,283	44	36.98	MH892372
SW28	21	Italy	UCCSt97	1	987	32,093	45	36.88	MH892374
SW4	26	Argentina	UCCSt89	1	5093	34,556	47	38.15	MH892355
SW19	9	Italy	UCCSt89	1	5093	35,153	46	38.10	MH892367
SW24	14	Italy	UCCSt89	1	5093	32,092	42	38.31	MH973663
SW27	21	Italy	UCCSt89	1	5093	33,953	45	38.25	MH892373

^†^ This number represents the number of strains infected by the phage in addition to the primary host. * These phage isolates are 100% identical to each other.

**Table 3:** Information associated with the samples used in this study.

Whey Sample Number	Country of Origin	Factory #	Starter Used ^ᴥ^	Sampling Year	Phage Positive	Types of Phages (# Propagated Isolates)	# Strains Infected	Phage Titre Range (pfu/mL)
1	Italy	1	DS1	2017	No	-	0	0
2	Italy	1	DS1	2017	No	-	0	0
3	Italy	1	DS1	2017	No	-	0	0
4	Italy	2	DS2	2017	No	-	0	0
5	Italy	2	DS2	2017	No	-	0	0
6	Italy	3	DS3	2017	Yes	*cos* (1)	1	10^2^
7	Italy	4	DS4	2017	Yes	987 (1)	1	10^2^
8	Italy	5	DS5	2017	Yes	*cos* (3), *pac* (2), 987 (1)	3	10^2^
9	Italy	6	DS6	2017	Yes	*cos* (16), *pac* (3), 5093 (1), 987 (1)	9	10^2^–10^6^
10	Italy	7	DS7	2017	Yes	*cos* (1)	1	10^2^
11	Italy	7	DS7	2017	Yes	*cos* (1)	1	10^2^
12	Italy	8	DS8	2017	Yes	*cos* (8), *pac* (1)	4	10^4^–10^6^
13	Italy	8	DS8	2017	Yes	*cos* (14), *pac* (1), 987 (2)	8	10^4^–10^9^
14	Italy	9	DS9	2017	Yes	*cos* (11), *pac* (2), 5093 (1), 987 (1)	8	10^3^–10^7^
15	Italy	9	DS9	2017	Yes	*cos* (15), 987 (1)	8	10^3^–10^7^
16	Italy	10	DS10	2016	Yes	*cos* (3)	2	10^2^–10^8^
17	Italy	10	DS10	2016	Yes	*cos* (12)	3	10^3^–10^5^
18	Italy	11	DS11	2016	No	-	0	0
19	Italy	12	DS12	2017	No	*-*	0	0
20	Italy	13	DS13	2017	Yes	*cos* (2)	1	10^7^
21	Italy	14	DS14	2016	Yes	*cos* (9), 5093 (2), 987 (2)	8	10^2^–10^6^
22	Spain	15	DS15	2017	Yes	*cos* (4)	3	10^6^–10^7^
23	Spain	15	DS15	2017	Yes	*cos* (5)	3	10^5^–10^7^
24	Spain	16	DS16	2017	No	-	0	0
25	Israel	17	DS17	2017	Yes	*cos* (3)	2	10^2^–10^4^
26	Argentina	18	DS18	2017	Yes	*cos* (2), 5093 (1)	2	10^4^–10^9^
27	Argentina	19	DS19	2017	Yes	*cos* (4) *pac* (1)	2	10^2^–10^3^
28	Turkey	20	DS20	2016	Yes	*cos* (16)	6	10^3^–10^7^
29	Turkey	21	DS21	2016	Yes	*cos* (6), *pac* (1)	7	10^2^–10^4^
30	Russia	22	DS22	2017	Yes	*cos* (1)	0	10^8^
31	Mexico	23	DS23	2016	Yes	*cos* (2)	1	10^2^
32	France	24	DS24	2016	Yes	*cos* (3)	2	10^3^–10^7^
33	Egypt	25	DS25	2016	No	-	0	0
34	Congo	26	DS26	2016	No	-	0	0
35	Austria	27	DS27	2017	Yes	*cos* (5)	1	10^3^

^ᴥ^ DS strains refer to defined starter cultures. Starters included defined *S. thermophilus* strains in all cases and incorporated strains of *Lactobacillus delbrueckii* ssp. *bulgaricus* (*Lb. bulgaricus*) in the case of DS3, DS15, DS21, DS22, DS25, and DS26. DS4 incorporated defined strains of *S. thermophilus*, *Lb. bulgaricus*, and *Lactococcus lactis*, while DS14 incorporated *S. thermophilus*, *Lb. bulgaricus*, *Lactobacillus helveticus*, and mesophilic strains. DS27 incorporated strains of *S. thermophilus* and *Lactobacillus delbrueckii* ssp. *lactis*.
